# Increasing Physical Activity at School Improves Physical Fitness of Early Adolescents

**DOI:** 10.3390/ijerph20032348

**Published:** 2023-01-28

**Authors:** Katarzyna Ługowska, Wojciech Kolanowski, Joanna Trafialek

**Affiliations:** 1 Faculty of Medical and Health Sciences, Siedlce University, 08-110 Siedlce, Poland; 2 Faculty of Health Sciences, Medical University of Lublin, 20-400 Lublin, Poland; 3 Institute of Human Nutrition Sciences, Warsaw University of Life Sciences, 02-787 Warsaw, Poland

**Keywords:** adolescents, Eurofit, fitness, physical activity

## Abstract

(1) Introduction: Regular physical activity (PA) is an important part of a healthy lifestyle for children and adolescents. The aim of this study was to assess the impact of an increase in organized PA at school on the physical fitness (PF) of early adolescent boys and girls. (2) Methods: A total of 294 children born in 2007 took part in the study. The sample was divided into two groups: of increased PA at school (*n* = 140, girls *n* = 66, boys *n* = 74) and standard PA (*n* = 154, girls G *n* = 70, boys *n* = 84). Increased and standard PA levels consisted of 10 and 4 h of physical education lessons (PE) per week, respectively. PE consisted of team games and fitness exercises. Three of the Eurofit tests, core strength, long jump, and running speed, were used to measure PF. Tests were conducted in May 2018 and 2019, at an average age of a participant of 11 and 12 years, respectively. Descriptive statistics and cluster analysis were applied for analyzing the results. (3) Results: After one year of observation PF of children was improved in both groups (*p* < 0.001). However, it was greater in the increased PA group than in the standard one. A higher percentage of very good scores and lower of poor and very poor were noted in the increased PA group than the standard one (20.36% vs. 12.90%, *p* = 0.003 and 18.58% vs. 24.85%, *p* = 0.022, respectively). Boys obtained better results than girls (*p* = 0.003). Children achieved the best results in the shuttle run test, and the worst in the core strength. Children with normal body mass obtained better results than those with excessive ones. (4) Conclusions: Increasing the number of PE at school beneficially affects the fitness performance of early adolescents. To improve the health status of adolescents it is advisable to increase the number of compulsory PE lessons in the school curriculum.

## 1. Introduction

Physical fitness (PF) is a good summary measure of the body’s ability to perform physical activity (PA) and exercises, and is an important health indicator. Regular participation in sports activities is an important form of PA for children and teenagers [1]. Often the terms PA, PF, and PF parameters are confused. WHO defines PA as any bodily movement produced by skeletal muscles that require energy expenditure and can be performed at varying intensities, while PF is defined as a set of attributes that are achieved or possessed in relation to the ability to perform PA or exercise with a specific skill affecting sports performance [2,3,4,5,6]. PF parameters are in other words maximum aerobic capacity, muscle strength, and flexibility [4].

One of the most common adverse behaviors among children and adolescents are sedentary lifestyle and low physical fitness. This leads to an increased risk of chronic diseases such as obesity, diabetes, cardiovascular diseases, renal failure, etc. [2,3]. One of the most promising strategies to minimize common health risks is increasing PA level and frequency. There are many actions taken around the World to encourage and promote PA among children and adolescents [3]. An adequate level of PA improves PF, which is one of the important indicators of health [2]. Both, the PA level and PF of children and adolescents should be monitored and measured [3]. Throughout the entire process of school education, motor skills are shaped in children. They have physical education lessons (PE) also aimed at encouraging children to take up PA later in life and the ongoing development of PF. 

In 2020, the World Health Organization (WHO) presented new recommendations on the PA level. For children and adolescents aged 5–17, 60 min of moderate to high-intensity PA daily is recommended [4]. There are a lot of desirable effects of frequent PA in adolescence such as improvement in skeletal and cardiovascular development and function, cardio-respiratory and muscular fitness and physical performance, as well as a positive impact on body weight [5,6]. In addition, it has been suggested that organized PA, especially team sports games and competitions, positively impacts the development of social behavior [4,7,8,9]. There is growing evidence of a link between physical inactivity and mental health decline. Sport provides a balance between group and individual demands and between aggressive behavior and self-control. Regular PA also leads to better circulation and oxygen supply to the brain, increased bone and muscle density, and greater tolerance to stress [4,8,9,10]. Previous studies also showed a positive effect of increased PA on body composition and blood pressure [10,11,12,13,14].

The WHO indicates that an adequate level of PA in childhood and adolescence plays an important role in preventing the global obesity epidemic [4]. Unfortunately, there is a global decline in general PA levels particularly in adolescence as well as an increase in sedentary behavior [15,16,17,18,19]. More than 80% of children and adolescents aged 11–17 years do not meet the recommendations for daily PA level [4,20,21,22,23]. There is a global problem of PF decline in school-aged children [4,20]. Also in Poland, low levels of PA and a decline in PF in children and adolescents were reported [21]. It was shown that only 21% of girls and 24% of boys at the age of 11 years had PA in line with WHO recommendations [22]. Only approximately 20% of adolescents participated in regular extracurricular forms of organized PA at least once a week [22,24,25]. Various programmes were being taken to counteract the decline in PA levels, e.g., the WHO Global Action Plan on Physical Activity (GAPPA) [26].

Reliable measurements of PF in children and adolescents are possible using standardized fitness tests. Currently, there are more than fifteen group tests for assessing PF worldwide [27]. The most commonly used include Eurofit, FitnessGram, and Unifittest [28,29,30]. The European Council’s Committee for the Development of Sport has created the European Fitness Test (Eurofit), which allows results to be compared between different countries using standardized methodologies [31,32]. Eurofit includes simple tests that can be performed as part of a physical education lessons (PE) [33,34]. PF parameters tested were agility, strength, muscular and cardiovascular endurance, flexibility, speed, and balance [31,32]. It was shown that the tests performed as part of Eurofit are reliable and the method introduced by Tomkinson et al. (2018) is used to interpret the results [35,36].

Adequate regular PA combined with a healthy diet are essential in maintaining good health from childhood to old age [37]. Schools should play an important role in promoting PA among children and adolescents. The aim of this study was to assess the impact of an increase in organized PA levels at school on the PF of early adolescent boys and girls. It was assumed that doubling the number of compulsory physical education lessons for a period of 12 months will significantly improve the PF of early adolescents.

## 2. Materials and Methods

### 2.1. Study Design

Participants were divided into two groups: of standard level of organized PA at school and an increased one. The first group gathered children for a standard number of physical education lessons (PE) (so-called general education classes—GC), which consisted of 4 h a week (the standard number of PE in primary school in Poland). The second group gathered children of an increased number of PE (so-called sports classes—SC), which consisted of 10 h a week. Children began their participation in the SC in the 4th grade of school. The SC did not focus on training a particular sport but on an increased curricular number of PE. PE consisted of team games and fitness exercises in organized form Six standard type primary schools in Siedlce (medium size city in Poland) took part in the study. In each school the GC and SC classes were organized were organized pararelly. From each school one SC and one GC took part in the study (in summary 6 CG and 6 SC). There were 22 to 25 children in each class.

### 2.2. Participants

The study was conducted on the same group of children born in 2007, between the ages of 11 and 12. A total of 294 children were tested, of which 154 GC (girls *n* = 70; boys *n* = 84) and 140 SC (girls *n* = 66; boys *n* = 74). The sampling strategy was convenience sampling. The number of children in each class resulted from the recruitment of candidates when enrolling children before the start of school. That is why selected groups were not equal. The a priori power analysis using the G*power software Ver. 3.1.9.7 (Kiel, Germany) indicated that number of 122 participants in the SC group and 123 in the GC (total 245) were sufficient to observe significant effects (Cohen’s d = 0.80) [38].

The exclusion criteria were as follows: (1) lack of consent of the parent or child to participate in the study; (2) poor health; (3) sick leave, (4) chronic diseases (e.g., cardiovascular diseases, cancer, respiratory diseases, diabetes, obesity, osteoporosis, autoimmune diseases, epilepsy, HIV/AIDS and chronic kidney disease); (5) newly diagnosed illness or hospitalization; (6) disability or injury to the lower limbs precluding PA; (7) absence from one of the measurement sessions; (8) diagnosed chronic diseases that could have affected the measurement result; (9) incorrectly performed PF tests; (10) resignation from performing tests during the study; (11) incorrect clothing and/or footwear. In addition, according to the adopted criteria, injuries, wounds, and malaise were among the factors excluding children from the fitness measurements. The parents declared that they would prepare the child for participation in accordance with the guidelines of the research team (described in Section 2.3). 

The project was approved by the Research Ethics Committee of the University of Siedlce (No. 2/2016). Parents gave their informed consent to their children’s participation in the study by completing the appropriate form. Parents and children were informed about the purpose of the study and the confidentiality of the results.

### 2.3. Procedure

The fitness tests were always performed in the gym [39]. At the time of testing, all participants were free of acute injuries and reported no musculoskeletal pain that could affect the test results.

The study was carried out by a research team of five licensed dietitians with the collaboration of PE teachers from the participating schools. Prior to the start of the study, team members were trained in the correct performance of the fitness tests, coding of data and confidentiality of results. The members of the research team knowingly and voluntarily agreed to participate in the study. Two measurement sessions were conducted and the results were systematically processed and analyzed. Measurements were taken in the presence of PE teachers. The first measurement session (initial) was conducted from 1 to 30 May 2018 and the second (final) from 1 to 30 May 2019. Fitness tests and anthropometric measurements were conducted in the same way in all schools. Each child was given an identification number, which was used throughout the study.

Children and their parents, school management and teachers were informed about the purpose of the study, the confidentiality of the results and declared their informed consent to participate in the study by signing a declaration to that effect. The management, parents and children received detailed information related to the date of the planned study two weeks earlier. This procedure allowed for high attendance throughout the study and proper preparation for the meetings. The date of the planned measurements was always agreed with the PE teachers and the school management. One month before the measurement, the parents received written information about the date of the measurement with a request to prepare the child for it. Other studies have shown a positive effect of parental involvement in measurement preparation [40]. Parents were asked to stop their children from eating a heavy meal after 21:00 the evening before the day of measurement and to serve them a light breakfast on the day of measurement. Just before the measurement, children were asked not to eat or drink until the measurement was completed. In addition, they were asked to refrain from significant physical exertion 24 h before the measurement, and they could perform their last activity at least 12 h before the measurement. The research team recommended that the last organized PA (for example, team games, competition, long-distance running) except for essential housework should not be performed at least 12 h before the measurement. The children had appropriate clothing prepared for fitness measurements (t-shirt with short sleeves, shorts) and sports shoes. The parents received the guidelines in paper form 7 days before the planned measurement and a reminder from teachers 4 days earlier.

The main study was preceded by a pilot study conducted among children at approximately 10 years of age (4th grade of primary school) *n* = 43 (GC 22; SC 21). The aim of the pilot study was to test the adopted methods and evaluate the procedures and measurement flow. The methods were properly planned and efficiently applied. The requirements for conducting a pilot study were similar to others of this type [41]. The main study used methods previously verified in the pilot study.

The interpretation of the results focused on the analysis of PF as measured by the three selected Eurofit tests in relation to body mass index (BMI). The results were systematically analyzed and made available to the parents during meetings once a semester.

### 2.4. Measurements

#### 2.4.1. Anthropometric Measurements

All measurements were carried out with uniform and standard equipment. Body height was measured in an upright position using a stadiometer brand SECA 214 with an accuracy of 1 cm. Body weight was measured with the Tanita SC-240MA device with an accuracy of 0.1 kg in children without shoes, in light clothes. Measurements of body weight and height made it possible to determine the BMI. The obtained values were related to the BMI charts for the population of Polish children. The range of BMI ≥ 85th percentile was assumed as overweight, BMI ≥ 95th as obesity, and BMI ≤ 10th as underweight.

The procedures of anthropometric measurements and the interpretation of the results were described in our previous papers [12,13,42].

#### 2.4.2. Eurofit Tests

Three of Eurofit tests were used to measure the PF of children in order: core strength, long jump, and running speed [31,32]. The tests were simple to carry out and could be organized in a school setting. The full Eurofit set of tests consists of 9 tests. However, it was impossible to perform all of them during one PE lesson. The choice of three tests used in the study was intentional, because the selected tests were usually performed as part of PE lessons and did not require any specialized equipment or apparatus. All tests were conducted by the research team with the assistance of PE teachers. Before starting the measurements, the study team prepared the necessary equipment (stopwatch, mattresses, measuring tape, and rubber cones). The study was conducted between 10 and 11 a.m. in the gym with a PE teacher following the guidelines described below [31,32,36]. At the beginning anthropometric measurements were carried out according to the methodology described in previous papers [10,12,13,42].

The Council of Europe guidelines and the standards developed by Tomkinson et al. (2018) were used to analyze the validity of the fitness tests (2018) [31,35,36]. In the core strength test (sit-ups in 30 s; *n*—number of sit-ups), the participants were asked to sit on a gymnastics mat with their back straight, hands behind their neck, elbows pointing forward, knees bent at 90° and feet flat on the mat. Then they were instructed to lie on their back with shoulders touching the mat and return to a sitting position with elbows pointing forward so that they touch their knees during the movement phase. If participants were unable to lift their backs off the floor, they scored 0 [31,36]. The number of correctly performed sit-ups in 30 s was recorded. The test was performed once. In the long jump test (long jump from a standing position, cm), participants stood behind the start line and had to perform a vigorous jump as far as possible and land with their feet together. The test was performed twice and the obtained score was the longest jump performed recorded to the nearest 0.1 cm. In a situation where the participant fell backwards, they had to repeat the jump [31,36]. In the running speed test (10 × 5 m shuttle run), two parallel lines were drawn on the floor 10 m apart, and participants were asked to run as fast as possible, crossing each line with both feet each time. The test was performed once, covering a distance of 50 m (5 × 10 m). When a participant overstepped the line, they had to repeat the test. The time was recorded to the nearest tenth of a second. The test was performed once, the score being the time taken to complete five cycles. Time was measured with a stopwatch [31,32,33,35,36].

Each test was demonstrated and explained verbally before being performed, and the children were given the opportunity to ask questions during the instruction. A 15-min warm-up led by the PE teacher was conducted before the tests were performed. Each participant took three tests with 5 min rest between them. The children were free to refuse the fitness tests at any time, and throughout the whole study period no one gave up or refused the tests.

Fitness test results were interpreted based on reference percentile curves for the Eurofit tests according to Tomkinson et al. (2018) [36]. A result corresponding to the 20th centile indicated a very poor score, between 20–40 a poor score, 40–60 a moderate score, 60–80 a normal score and ≥80 a very good score [36]. The results were systematically processed and analyzed in Microsoft Excel (Microsoft, Intentional Software, Washington, DC, USA) [43].

In the assessment of the children’s general PF (Section 3.4) between the initial and final measurements, the averages of the Eurofit tests analyzed over the entire study period were used.

### 2.5. Statistical Analysis

Statistical calculations were performed using Microsoft Excel 365 (Microsoft, Intentional Software, Washington, DC, USA) and the Statistica 13 software (Stat Soft, Kraków, Poland) [43,44]. The adopted level of significance was α ≤ 0.05.

Descriptive statistics allowed the determination of the average, median, standard deviation and 95% confidence interval at group, gender, and age levels and for each of the Eurofit tests. The Shapiro-Wilk test, the Mann–Whitney U Test, and Fisher–Snedecor test (F; one-way ANOVA analysis of variance) were performed. The Mann-Whitney U Test compared the criteria for assessing children’s PF (separately for each test) by group type (SC, GC) and gender, as well as in relation to body mass index. The Mann-Whitney U Test was also used to analyze children’s general PF by class profile (GC, SC) and gender, and to assess differences between initial and final measurements. The ANOVA was used to examine the effect of school PA level on average Eurofit test scores between group type (SC and GC) and gender. It was performed after checking the normality of the distribution and the homogeneity of the variance. Analysis of Covariance (ANCOVA) was applied to evaluate differences between initial and final measurements. The normality of the distribution was checked using the Shapiro-Wilk test, and the assumption of homogeneity of variance was verified using Levene’s Test, for *p* > 0.05, i.e., values greater than the accepted significance coefficient equal to 0.05 [44]. Effect sizes (ES) for the average fitness score obtained were calculated based on Cohen’s d. The threshold values for the ES statistics were as follows: >0.2 low, >0.5 moderate, >0.8 high, >1.3 very high [45].

In addition, a multi-dimensional cluster analysis was used to assess the influence of gender on the results of sports performance. A multi-dimensional cluster analysis calculation was performed to build a tree diagram where the fitness test results obtained by girls and boys from both SC and GC groups were most similar in a specific cluster. It used internal measures for cluster validation, as the matrix and Euclidean distance. The Ward method, as a hierarchical clustering method, was used to create groups, where the variance within the groups is minimized [44]. 

## 3. Results

### 3.1. Description of the Group

Children were approximately 10.90 years old at the start of the study (SC 10.89; GC 10.90) and 11.90 at the end of the study (SC 11.89; GC 11.90). Test results were obtained from 294 children (girls 46%; boys 54%). The number of participants in both groups was similar (SC *n* = 140; GC *n* = 154). In the increased PA group (SC), 140 children were tested, (girls *n* = 66, boys *n* = 74). However, due to not meeting the criteria (i.e., joining the class during the study), approximately 2% of participants (*n* = 3) (girls 1.5%; boys 2.70%) were not included in further analysis. Results from 137 SC children who attended all measurement sessions (girls *n* = 65; boys *n* = 72) were included in the final analysis. In the standard PA group (GC), 154 children were tested (girls *n* = 70; boys *n* = 84). Results from 135 GC children who attended all measurement sessions were included in the final analysis (girls *n* = 65; boys *n* = 70). Finally, the analysis included results of a total of 272 children (SC *n* = 137; GC *n* = 135; *p* = 0.278), including 48% girls (*n* = 130) and 52% boys (*n* = 142).

### 3.2. Eurofit Tests

#### 3.2.1. Core Strength

According to the reference curves for the Eurofit tests, the expected score (≥normal) of the sit-ups in 30 s for girls aged 11 is ≥17, for boys aged 11 ≥ 19 [36]. In the core strength test, SC children performed significantly better than GC children. Regardless of gender or group profile, there were better results in the final session than in the initial (20.62 vs. 18.67; *p* < 0.001). A significant increase in the number of sit-ups performed during testing was observed in both the GC and SC groups (GC from 18.00 to 19.99; *p* = 0.001; SC from 19.31 to 20.34; *p* = 0.022), with a significantly higher number in the SC than GC (Table 1).

On average, the SC girls performed slightly better than GC, also the maximum values were more frequently observed in the SC. On average, girls in both groups managed to meet the required number of sit-ups. The increase in the number of sit-ups during the study was 1.91 (GC 1.9; SC 1.93; *p* = 0.065). On average, underweight girls performed 19.47 sit-ups (GC 18.50; SC 20.44; *p* < 0.001), girls with normal body weight 20.20 (GC 19.30; SC 21.10; *p* = 0.050), overweight girls 16.56 (GC 15.67; SC 17.45; *p* < 0.001) and obese girls 13.45 (GC 13.04; SC 13.87; *p* = 0.003). In all weight categories, a significantly better average score was achieved by the SC girls than GC ones.

On average, the SC boys had slightly better scores than GC (Table 1). All participants were within the normal ranges of the reference curves. The increase in the number of sit-ups from the initial session to the final session was on average 1.98 (GC 1.91; SC 2.06; *p* < 0.001). Underweight boys performed 21.66 sit-ups (GC 21.02; SC 22.30; *p* = 0.002), boys with normal body weight 22.15 (GC 21.80; SC 22.51; *p* = 0.003), overweight boys 17.80 (GC 17.40; SC 18.19; *p* = 0.025) and obese boys 14.32 (GC 13.48; SC 15.17; *p* < 0.001). In all weight categories, a significantly better average score was achieved by the SC boys than GC.

Interpretation of the results based on the Eurofit reference curves showed that, on average, 44.88% of children had a normal number of sit-ups in 30 s (GC 44.67%; SC 45.08%; *p* = 0.005). However, a very good score was significantly more frequently achieved by the SC children than GC (SC 19.68%; GC 12.32%; *p* < 0.001). A poor score was significantly more frequently achieved by the GC children than SC (GC 17.19%; SC 13.11%; *p* = 0.002).

**Figure 1:**
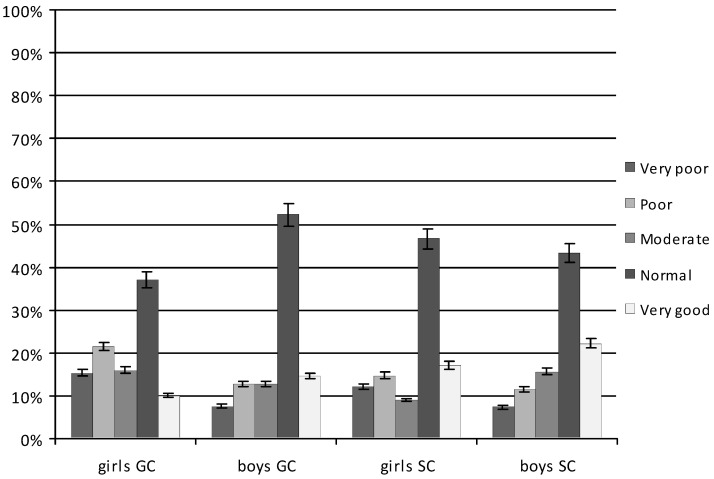
Results of the 30 s sit-up test, an average over the study period. GC—general education classes; SC—sport classes.

In girls, an average of 42% performed the normal number of sit-ups (GC 37.10%; SC 46.72%; *p* < 0.001). The SC girls performed significantly more sit-ups than GC, and they significantly more often than GC achieved a very good score (17.21% and 10.05%; *p* = 0.005, respectively). An average of 18.31% of the girls achieved a poor score, with GC significantly more often than SC (GC 21.50%; SC 14.76%; *p* < 0.001). Nearly 14% achieved a very poor score, more often GC than SC (GC 15.35%; SC 12.29%; *p* = 0.003) (Figure 1).

On average, almost 50% of boys achieved a normal score, with SC significantly more often than GC (GC 43.44%; SC 52.23%; *p* < 0.001). The SC boys were significantly more likely than GC to obtain a very good score (SC 22.14%; GC 14.60%; *p* < 0.001) and GC boys to obtain a poor score (GC 12.88%; SC 11.47%; *p* = 0.023). A very poor score was achieved by a similarly small percentage of boys in both groups (GC 7.57%; SC 7.37%; *p* = 0.065).

In the sit-up test, boys got better scores on average than girls. A very good score was recorded among 13.63% of girls and 18.37% of boys (*p* < 0.001). Poor and very poor scores were recorded among 18.13% and 13.82% of girls and 12.18% and 7.47% of boys, respectively (*p* < 0.001).

#### 3.2.2. Long Jump

The long jump test showed that the SC children obtained better results than GC (Table 2). In the final measurement session, the children performed on average approximately 10 cm better compared to the initial session (GC 9.44 cm; SC 10.41 cm; *p* < 0.001).

In general, girls performed on average better in the final measurement session than the initial one and improved their jump by an average of 9.22 cm (GC 9.21 cm; SC 9.23 cm; *p* = 0.750). The minimum values were the same in both groups, while the longest jump was recorded in the SC girls (Table 2). Underweight girls achieved an average score of 144.77 cm (GC 143.03 cm; SC 146.51 cm; *p* = 0.003), girls with normal body weight 144.72 cm (GC 142.10 cm; SC 147.34 cm; *p* < 0.001), overweight girls 129.32 cm (GC 124.18 cm; SC 134.46 cm; *p* = 0.001) and obese girls 122.09 cm (GC 120.38 cm; SC 123.80 cm; *p* = 0.048). In all weight categories, a significantly better average scores were achieved by the SC girls than GC.

Over the course of the study, the boys improved their long jump score by an average of nearly 11 cm (GC 9.67 cm; SC 11.59 cm; *p* < 0.001) (Table 2). The SC boys jumped significantly farther than GC. On average, underweight boys made a jump of 152.42 cm (GC 151.42 cm; SC 153.42 cm; *p* = 0.024), boys with normal body weight 155.25 cm (GC 151.45 cm; SC 159.06 cm; *p* = 0.015), overweight boys 141.26 cm (GC 140.60 cm; SC 141.92 cm; *p* = 0.037) and obese boys 122.03 cm (GC 121.10 cm; SC 122.97; *p* = 0.005). In all weight categories, a significantly better average scores were achieved by the SC boys than GC boys.

**Figure 2:**
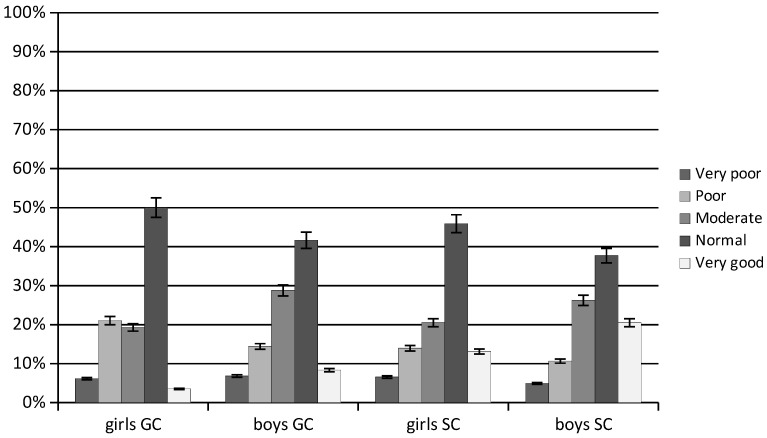
Results of the long jump test from a standing position, an average over the study period. GC—general education classes; SC—sport classes.

The expected long jump test score for girls aged 11 according to percentile reference curves for the Eurofit tests is ≥146.00 cm and for boys 157.70 cm. On average, more than 11% of children achieved a very good score, with the SC significantly more often than GC (GC 5.92%; SC 16.80; *p* = 0.000%) (Figure 2). Nearly 45% of the children achieved a normal score (GC 45.83%; SC 41.80%; *p* = 0.052). A moderate score was achieved by nearly 25% of children (GC 24.04%; SC 23.36%). Poor and very poor scores were significantly more frequently recorded in the GC than SC (17.73% and 6.48% vs. 12.30% and 5.74%; respectively, *p* < 0.001). 

On average, nearly 50% of girls achieved a normal long jump score (GC 50%; SC 45.90%; *p* = 0.023). Very good values were only recorded in 8.31% of girls, significantly more often in the SC girls than GC (GC 3.51%; SC 13.11%; *p* < 0.001). A poor score was significantly more frequently recorded in the GC than SC (21.05% vs. 13.94%; *p* < 0.001), and a very poor score was achieved by nearly 6.5% of girls (GC 6.14%; SC 6.56%; *p* = 0.055).

On average, 40% of boys achieved a normal long jump score (GC 41.66%; SC 37.70%; *p* = 0.550). A very good score was significantly more frequently achieved by the SC boys than GC (GC 8.33%; SC 20.49%; *p* < 0.001). Poor and very poor scores were significantly more frequently achieved by the GC boys than SC (GC 14.40% and 6.82%; SC 10.66% and 4.92%; respectively; *p* = 0.003).

The normal long jump score was achieved by 48% of girls and 39.68% of boys. A very good score was significantly more frequently achieved by boys than girls (14.41% and 8.31% respectively; *p* < 0.001). Girls were more likely than boys to achieve a moderate score (girls 27.51%; boys 19.89%; *p* = 0.002). 

#### 3.2.3. Shuttle Run 10 × 5 m

According to the percentile grids for the agility test, i.e., the 10 × 5 m shuttle run, the normal range for girls aged 11 is ≥ 22.04 s, and for boys ≥ 21.33 s [36]. Table 3 shows the results obtained in the agility test. The range of scores in the GC group was 17.22–32.00 s and SC 18.01–29.03 s On average, the SC children showed better results than GC (21.56 and 22.07, respectively; *p* = 0.003) (Table 4). Comparing the results from the initial and final measurement sessions, there was a significant improvement in scores in the GC group by 1.02 s and SC by 1.20 s (*p* = 0.024).

The girls’ average shuttle run time was 22.30 s. (GC 22.52; SC 22.09; *p* = 0.035) (Table 3). Comparing the results from the initial and final sessions, the girls improved their score in the GC by 1.01 s, in SC by 1.09 s (*p* = 0.037). On average, better results were recorded in the SC than GC. Underweight girls achieved an average time of 21.51 s (GC 21.73 s; SC 21.30 s; *p* = 0.004), girls with normal body weight 21.22 s (GC 21.55; SC 20.89; *p* < 0.001), overweight girls 23.46 s (GC 24.28; SC 22.64; *p* = 0.003), and obese girls 24.94 s (GC 25.65; SC 24.23; *p* < 0.001). In all weight categories, a significantly better average scores were achieved by the SC girls than GC ones.

The average score in the agility test in boys was 21.31 s (GC 21.61; SC 21.02; *p* = 0.150) (Table 3). Comparing the results from the initial and final measurement sessions, the boys improved their running time by its reduction average 1.17 s (GC 1.03; SC 1.32; *p* = 0.005). The range of test scores was 18.73–32.00 s in the GC group and 18.01–24.98 s in SC. Boys with normal body weight obtained an average score of 20.85 s (GC 20.96; SC 20.75; *p* = 0.670), underweight boys 21.19 s (GC 21.24; SC 21.15; *p* = 0.450), overweight boys 22.40 s (GC 22.41; SC 22.39; *p* = 0.132), and obese boys 24.40 s (GC 24.85; SC 23.99; *p* = 0.120). In all weight categories, better average scores were achieved by the SC boys than GC ones. Statistical analysis, however, did not show significant differences in the shuttle run test.

An average of 43% of children achieved a normal shuttle run score (GC 42.40%; SC 43.44%; *p* = 0.052). The SC children were significantly more likely than GC to obtain a very good score (SC 24.60%; GC 20.46%; *p* < 0.001) and normal score (GC 42.40%; SC 43.44%; *p* = 0.032). Poor and very poor scores were significantly more frequently achieved by the GC children than SC (GC 15.89% and 5.78%; SC 13.11% and 1.64; respectively; *p* = 0.002 and *p* < 0.001).

**Figure 3:**
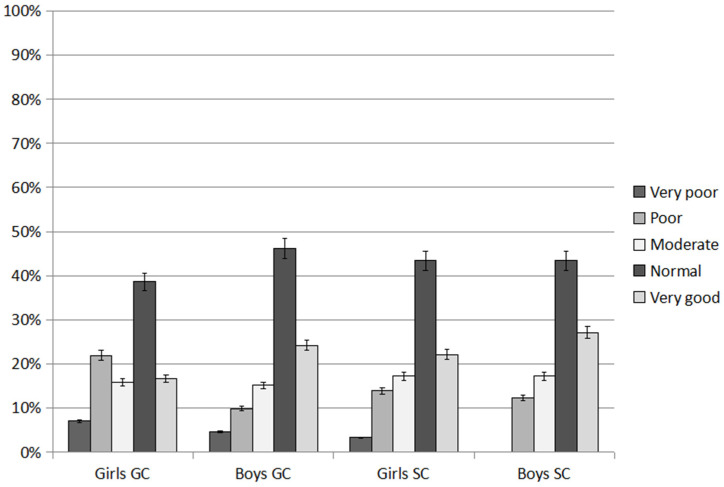
Results of the 10 × 5 m shuttle run test, an average over the study period. GC—general education classes; SC—sport classes.

On average, 41% of girls achieved a normal shuttle run test score (GC 38.59%; SC 43.44%; *p* = 0.003) (Figure 3). The SC girls were significantly more likely than GC to achieve a very good score (22.14% and 16.67%; respectively; *p* = 0.002). The GC children were significantly more likely than SC to achieve a poor score (GC 21.93%; SC 13.93%; *p* < 0.001). A very poor score was noted among 5.15% of girls, significantly more often in the GC than in SC (GC 7.02%; SC 3.28%; *p* = 0.004).

On average, 45% of boys achieved a normal shuttle run score (GC 46.21%; SC 43.44%; *p* = 0.003) (Figure 3). A very good score was achieved by 27.06% of the SC boys and 24.24% GC (*p* < 0.001). A moderate score was achieved by 16.19% of boys (GC 15.16%; SC 17.21%; *p* = 0.002). None of the SC boys scored very poorly, with 5% in GC.

On average, boys were significantly more likely to obtain a very good score (25.65%) and a normal score (44.83%) than girls (respectively: (19.41% and 41.02%; *p* < 0.001, respectively). Girls were significantly more likely to score poor (17.93%) and very poor (5.15%) than boys (respectively: (11.07% and 2.28%; *p* < 0.001, respectively).

### 3.3. Averaged Results

The averaged results of fitness tests are shown in Table 4. Significant differences were observed between the GC and SC groups. Over the study period, the SC group performed significantly better than GC. Minimum values were more frequently recorded in the GC group, and maximum values in SC. Statistical analysis showed significant differences between the average test results between the GC and SC groups. 

Over the study period, boys showed higher fitness levels than girls (Table 5). Maximum values were significantly more frequently recorded in boys and minimum values in girls. Statistical analysis showed significant differences between the performance of girls and boys in each fitness test.

**Figure 4:**
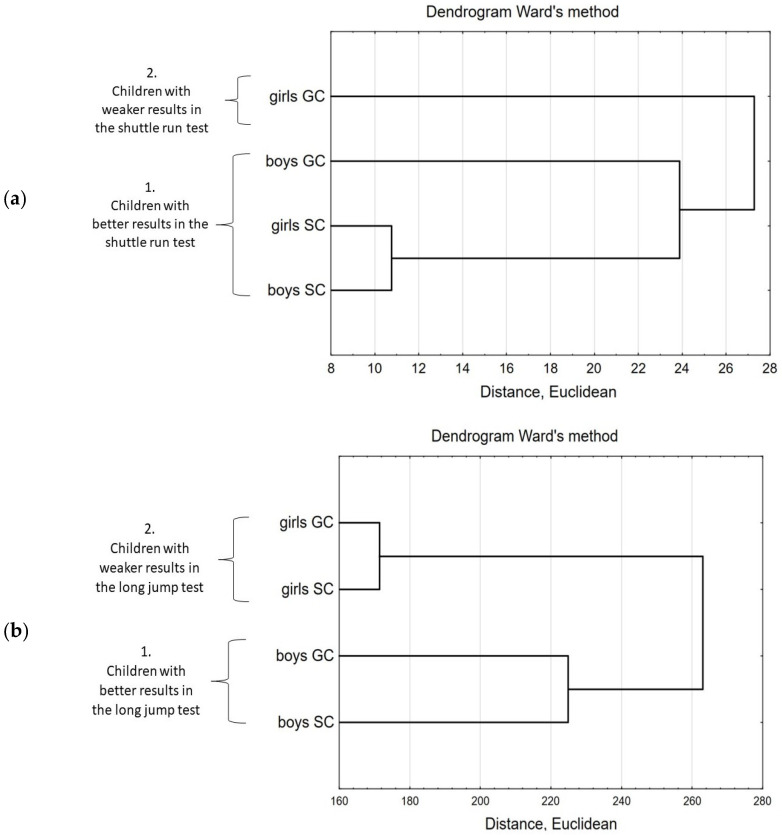

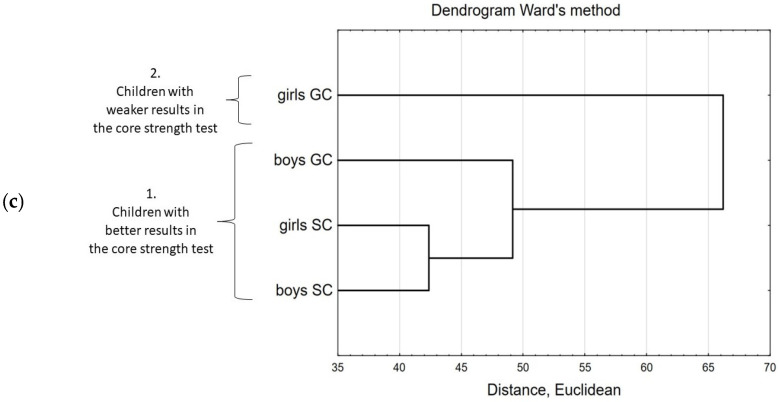
The cluster analysis of SC and GC boys and girls fitness tests results; separated clusters showed groups of similar results in particular tests: (**a**) shuttle run test; (**b**) long jump test; (**c**) core strength test. GC—general education classes; SC—sport classes.

The impact of gender on the fitness test results was deepened by multidimensional cluster analysis (Figure 4). Separated clusters showed groups of similar results in particular fitness tests. In the case of shuttle run test, two clusters were created. The first one (cluster 1) contains SC boys and girls as well as GC boys who achieved significantly better results than GC girls (cluster 2). In the case of long jump test, two clusters were created. SC and GC boys achieved significantly better results (cluster 1) than SC and GC girls (cluster 2). In the case of core strength test, SC boys and girls as well as GC boys achieved significantly better results (cluster 1), than GC girls (cluster 2). 

### 3.4. General Physical Fitness

**Figure 5:**
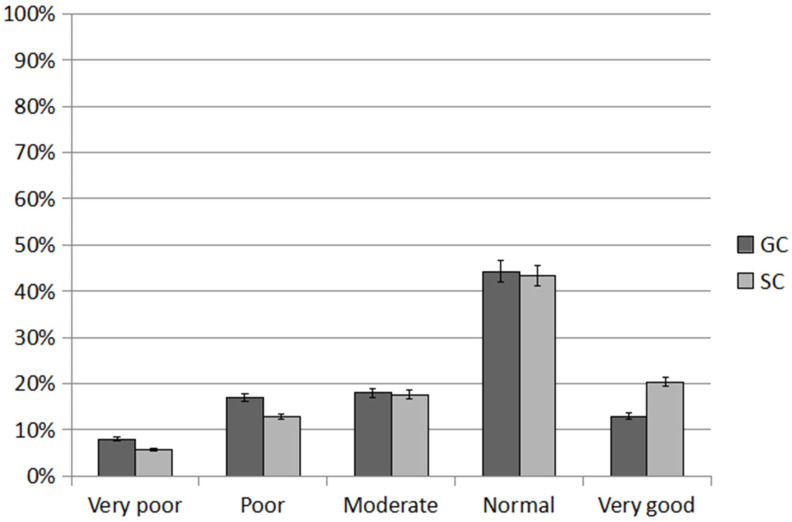
General physical fitness, the average of all fitness tests results. GC—general education classes; SC—sport classes.

In the assessment of general PF, scores from all fitness tests were averaged. The SC group had a higher percentage of very good results than GC (20.36% and 12.90%; respectively; *p* = 0.003). Poor and very poor results were recorded more frequently in the GC group than SC (24.85% and 18.58%; respectively; *p* = 0.022). The SC children scored nearly 44% normal: 21% very good and 18% moderate (Figure 5). Approximately 13% of children had poor performance and 5.74% had a very poor one. In the SC group, a very good score was most frequently obtained in the shuttle run test (24.60%), while poor and very poor scores were most frequently recorded in the core strength test (13.11% and 9.83%; respectively). In the long jump test from a standing position, almost 42% of the children achieved a good score and 24% a moderate score.

In the GC group, an average of 44.30% of children had a normal score, about 18% moderate, 16.93% poor, and 7.90% very poor. A very good score was recorded only among 12.90% of GC children. The highest percentage of normal scores in the GC group was recorded in the long jump test (45.83% of children), while very good scores in the agility test (20.46%). The poorest scores (poor and very poor) in the GC group were recorded in the core strength test (respectively: 17.19% and 11.46% of children).

**Figure 6:**
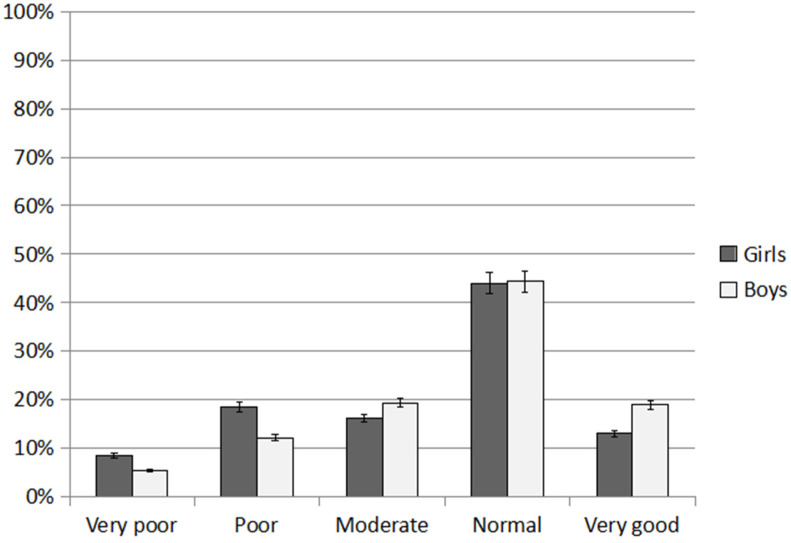
General physical fitness of girls and boys, an average of all fitness test results.

Analyzing PF in relation to gender it was shown that boys were characterized by better fitness than girls (Figure 6) A very good score was recorded in nearly 13% of girls and 19% of boys (*p* = 0.003). A moderate score was recorded in 43.97% and 44.36% respectively (*p* = 0.140). Poor and very poor scores were recorded more frequently among girls than boys (13.42% vs. 8.70%; *p* < 0.001). Moderate values were reported in 16.20% of girls and 20% of boys (*p* = 0.035).

In summary, children’s PF in relation to the percentile reference curves for the Eurofit tests was mostly normal (GC 44.30%; SC 43.44%; *p* = 0.055). Nearly 17% of children were characterized by very good scores, significantly more often in the SC group than GC (GC 12.90%; SC 20.36%; *p* < 0.001). A moderate score was achieved by nearly 18% of children (GC 17.96%; SC 17.63%; *p* = 0.120). A poor score was achieved by 14.89% (GC 16.94%; SC 12.84%; *p* < 0.001) and a very poor score by 6.82% (GC 7.91%; SC 5.74%; *p* = 0.023). 

**Figure 7:**
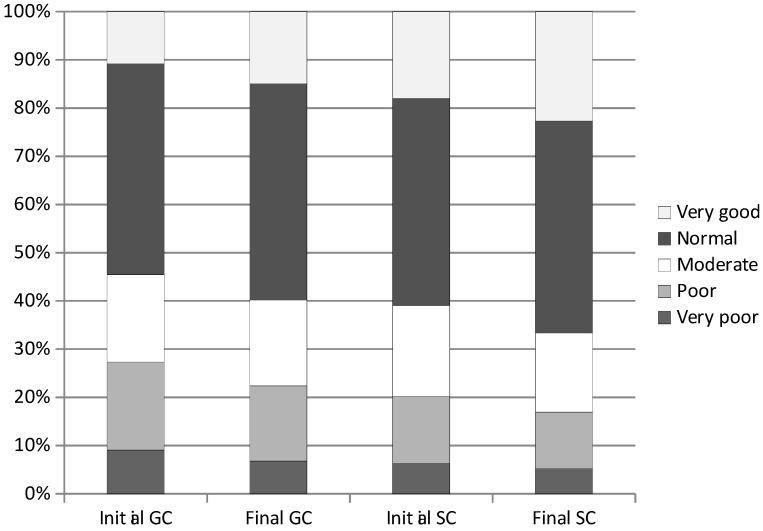
Comparison of children’s general physical fitness between the initial and final measurements. GC—general education classes; SC—sport classes.

It was observed that, compared to the initial measurement, in the final measurement children performed better on average on each of the tests. Also, the interpretation of average scores in relation to the percentile curves indicated an improvement in fitness performance towards the end of the study (Figure 7). The percentage of very good general PF scores was highest in the final session in both the GC group and SC (14.96%; 22.68%; respectively; *p* < 0.001). Similarly normal scores (GC 44.86%; SC 43.98%; *p* < 0.001). The percentage of normal scores increased in GC from 43.74% to 44.86% (*p* = 0.076) and in SC from 42.90% to 43.98% (*p* < 0.001). The percentage of moderate scores decreased from 18.12% to 17.80% (*p* = 0.203) in the GC group, and in SC from 18.85% to 16.40% (*p* < 0.001). There was a decrease of poor and very poor scores, particularly in the SC group from 20.21% to 16.94% (*p* < 0.001), and in GC from 27.31% to 22.38% (*p* < 0.001).

## 4. Discussion

Participation in organized forms of PA is a good way to promote a healthy lifestyle [46]. The current level of PE lessons in the primary school curriculum in Poland (4 h a week) does not correspond to the WHO recommendations for PA [4]. In this study, it was observed that the children achieved, on average, better results in each of the assessed tests in the final measurement compared to the initial. However, this improvement was significantly greater in the PA-increased group than the standard one. In all tests, children with higher than standard PA performed better. Boys performed better than girls. More than half of the children in the study obtained normal scores on fitness tests, and 17% obtained a very good score. The most frequent scores were very good in the shuttle run test, poor and very poor in the core strength test. A moderate score was most often achieved in the long jump test. Better scores were reported in the study by Puchalska-Sarna et al. (2022) [47]. In this study, 58% of physically active children achieved PF tests results similar to those presented in the present study. Similar observations were made by Abdelkarim et al. (2019) [48], Díaz (2019) [49], and Fiori et al. (2020) [50]. Research confirms the relationship between a child’s higher PA and better sports performance [51]. This was also shown in the present study.

Improvements in children’s fitness performance were noted with the duration of increased PA at school. The study by Puchalska-Sarna et al. (2022) conducted in a group of children aged 11–15 also shows age-related improvements in PF in children [47]. There is also evidence to suggest otherwise, for example, the study by Weedon et al. (2022) reported a downward trend in sports performance in children at the time of the study [52]. This is due to the general increasing trend of a sedentary lifestyle among adolescents. However, increasing the number of compulsory PA at school can counteract it to some extent, haw it was shown in the present study.

On average, boys performed better than girls in each of the tests. Such results are not surprising; in fact, biological maturity exerts a decisive influence on the PF of boys and girls [53]. Although there are no differences in pre-adolescent stages they appear at around 9–15 years of age in girls and 12–16 years in boys [54,55]. This age often causes changes in PF, as well as regression of some motor skills. It is related to the change of hormones, lowering of self-esteem, lack of acceptance of the body, low PA and change in BMI [18]. Similarly, Riddoch et al. indicate that European studies of children’s PF showed higher levels of fitness in boys than in girls [56].

Boys in particular scored higher in the 5 × 10 m shuttle run than girls. Also, in a study by Gea-García et al. (2020) boys scored higher on tests similar to those presented in this study [14]. The opposite was observed in the study by Mancini et al. (2022) conducted on children aged 10–13, which reported no differences in the speed test between physically active boys and those not undertaking additional PA [57]. However, it was a pilot study and long-term outcomes were not monitored.

In the present study, in general, girls performed more poorly in the core strength test than boys. However, SC girls performed similarly to SC boys and even slightly better than GC boys. In the study by Tomkinson et al. (2018) conducted with the Eurofit test showed that boys performed significantly better than girls in tests of muscular strength, muscular endurance, and speed but worse in the core strength test [36]. Also in the study by Mancini et al. (2022), girls showed better fitness performance in the core strength test than boys [57].

It is well known that overweight and obesity result in a decrease in sporting ability [14,57,58]. In fact, motor skills such as running, jumping, or sit-ups are often hampered by excessive body weight. The results of the present study showed that overweight and obese children had worse fitness performance, which was consistent with other studies [59,60,61,62]. In the present study, overweight children performed a long jump with an average length of 135.29 cm, and obese children—122.06 cm, also the scores in the speed test were significantly lower than in the children with normal weight amounting to 22.93 s for overweight children, and 24.67 s for obese ones. Slightly better scores were obtained by Vandoni et al. (2021) [58]. In the study cited above, overweight children performed a long jump from a standing position of 137.57 cm, and obese children of 139.63 cm, and to complete the speed test, i.e., a 5 × 10 m shuttle run, they needed 22.09 s, and obese children needed 22.15 s [58]. Other authors also indicated that children with excessive body weight had difficulty performing exercises such as shuttle runs and long jump tests [59,60]. The study by Morano et al. (2020) showed that an additional cycle of seven months of sports activities for overweight and obese children aged 10–12 had beneficial effects [61]. Furthermore, it was shown that overweight children undertaking additional PA achieved better scores than overweight and obese peers without PA intervention [61]. In the study by Kwieciński et al. (2018) on the PF assessment of normal and overweight (overweight and obese) adolescents showed that fitness test scores differed according to BMI [62]. In the shuttle run and long jump tests, boys with a normal BMI performed best, while underweight, overweight and obese children performed worse [62].

On average, the children had the poorest scores in the 30 s sit-up test. It was observed that they performed almost 19 sit-ups at the beginning of the study and almost 20 after one year of follow-up. The girls performed close to 19 and the boys almost 21. Poorer scores prior to the introduction of the PA intervention were reported in the study by Fransen et al. (2014) [63]. In the study cited above, the children performed an average of 12 sit-ups- and 18 sit-ups after 2 years of follow-up and intervention associated with increased PA. Similarly, in the present study children had a better score after 1 year of intervention compared to children without additional PA. This demonstrates the beneficial role of regular and organized PA at school on fitness performance. In the study of Korcz and Monyeki (2018) among older children (14 years old) the results of the 30 s sit-up test also showed more favorable scores among participants involved in additional sports activities compared to children not undertaking extracurricular or additional PA (29.19 vs. 22.57) [64]. Similarly, in the present study, both girls and boys with increased PA at school performed more sit-ups compared to peers without additional PA [64]. The study by Dobosz et al. (2015) showed on average higher fitness performance in boys than girls, with the exception of tests of balance and core strength, in which girls performed slightly better [65]. Studies by Zhang et al. (2021) [66] and Mancini et al. (2022) [57] also indicated better scores in the core strength test in girls, as already discussed above.

Analysis of the fitness test results in the present study showed that children performed best in the agility test, i.e., the 10 × 5 m shuttle run. On average, children with increased PA at school achieved more favorable scores in the agility test than those of standard PA (21.56 s and 22.07 s; respectively). Similarly, in the study of Mancini et al. (2022) children with additional PA needed 20.9 s, while those in the standard group needed 21.20 s [57]. In the study of Fiori et al. (2020) girls aged 10 performed the shuttle run in 23.4 s, aged 11 in 23.0 s, and boys in 21.90 s and 20.73 s, respectively [50]. Gea-García et al. (2020) showed that children aged 11.7 needed approximately 21.50 s to complete the test [14]. These results were similar to the present study.

Children in the increased PA group were characterized to a greater extent by good and very good scores of long jump performance than the standard one (16.80% vs. 5.92%). In the study of Martinez-de-Quel et al. (2021) boys aged 12–13 participating in sports activities performed a longer jump compared to the present study (198.4 cm vs. 155,24 cm, respectively) [67]. Also, the 12-year-old girls participating in the above study performed a longer jump (175.9 cm) compared to the present study (145.34 cm) [67]. However, these were young athletes, unlike those in the present study, who had an increased amount of PE. Orntoft et al. (2018) reported a jump of nearly 10 cm longer for children undertaking additional PA compared to those not undertaking additional PA [68].

On average, the children showed normal scores in the shuttle run test, moderate scores in the long jump test from a standing position and slightly too poor scores in the sit-up test. When comparing the scores from the initial and final measurements, the children improved their performance in all fitness tests regardless of gender and PA level at school. Children in the SC group scored better in all tests in both the initial and final measurements than GC ones. Overweight and obesity were associated with poorer fitness performance. Children with excessive BMI (overweight and obese) performed worse than those with a normal BMI, especially girls.

The results of the present study are in line with other reports showing differences in fitness scores depending on PA level [10,51,69]. Participation in organized forms of PA has lasting benefits that are considered attributes of a healthy lifestyle, but 4 h of PE lessons a week are not sufficient to maintain adequate PA levels in children and adolescents of sedentary behavior. Participation in additional compulsory PA classes during school time is a potential way to increase the PA level of school-aged children decreasing widely spreading sedentary behavior. In this way, children and adolescents forced to increase their PA at school could reach the WHO recommendations for PA level. An additional condition influencing children’s PF, besides the number of PE lessons at school, is the frequency of PA undertaken during leisure time. It was also measured and described by the authors of this study in another paper [70]. It was observed that, starting from a similar level in both GC and SC groups the frequency of PA during leisure time increased in the SC and decreased in GC during the course of the study.

This study has some limitations. The socio-economic level of children’s families was not characterized in the study. This was due to the possible high risk of lack of orientation about the real economic level of families among early adolescents. The Eurofit set of tests consists of nine tests, however, this study was limited to only 3 selected fitness tests. It was impossible to perform all of them in 21–24 children classes during one PE lesson. The study was limited to only a sample of children between the ages of 11 and 12. Further studies were planned, but due to the outbreak of the COVID-19 pandemic, this was not possible. Children’s extracurricular sporting activities was assessed only in part as the frequency of leisure time PA. The results were presented in a previous paper [70]. However, the direct and detailed measurement of sport activities during leisure time was not measured. The frequency of attendance at PE classes was also not analyzed due to measurements were conducted only twice. Moreover, PA levels and sporting achievements are also significantly influenced by the lifestyle of the children’s parents, but this was not studied due to a lack of parental consent. 

## 5. Conclusions

Increasing organized PA during school time through additional PE lessons improved the results of fitness tests of early adolescents. After one year of observation, there was a much higher improvement in fitness performance in the group with increased PA at school (SC) than in the standard one (GC). Generally, boys had better scores on fitness tests than girls. Children mostly were the best at the shuttle run test, and poor at the core strength test. Better fitness test results obtained for children with normal BMI than excessive body mass. Increasing the level of organized PA at school beneficially affects the physical fitness of early adolescents. To improve the health status of early adolescents it is advisable to increase the number of PE lessons in the school curriculum, as well as education about the health benefits of frequent PA.

## Figures and Tables

**Table 1:** Results of the core strength test, number of sit-ups in 30 s.

Measurement Session	Average Age (y/o)	Total Average (*n*/30 s)	Group												ESs	*p* *
			GC						SC							
			Average	Median	Min.	Max.	SD	95% CI	Average	Median	Min.	Max.	SD	95% CI		
Mean																
I	10.90	18.67	18.00	19.00	2.00	24.00	3.53	3.14–4.04	19.35	20.00	9.00	27.00	3.86	3.42–4.41	0.250	<0.001
II	11.90	20.62	19.90	21.00	4.00	26.00	3.50	3.11–4.00	21.34	22.00	13.00	29.00	3.67	3.26–4.20	0.614	0.010
Girls																
I	10.90	17.63	17.01	17.00	8.00	22.00	2.75	2.32–3.37	18.26	20.00	9.00	25.00	3.88	3.50–4.52	0.340	0.040
II	11.90	19.55	18.91	19.00	10.00	24.00	2.83	2.39–3.47	20.19	21.00	13.00	27.00	3.42	3.05–3.93	0.510	0.030
Boys																
I	10.90	19.70	18.96	20.00	2.00	24.00	3.88	3.31–4.69	20.44	22.00	12.00	27.00	3.53	3.00–4.30	0.560	0.020
II	11.90	21.68	20.87	22.00	4.00	26.00	3.78	3.23–4.56	22.50	23.00	14.00	29.00	3.48	2.95–4.24	0.240	0.060

GC—general education classes; SC—sport classes; measurement sessions: I (initial)—1–30 May-2018, II (final) 1–30 May-2019; Min.—minimum; Max.—maximum; SD—standard deviation; 95% CI—confidence interval; ESs—Effect sizes; * Mann-Whitney U Test; significant differences *p* ≤ 0.05.

**Table 2:** Results of the long jump test from a standing position, cm.

Measurement Session	Average Age (y/o)	Total Average (cm)	Group												ESs	*p* *
			GC						SC							
			Average	Median	Min.	Max.	SD	95% CI	Average	Median	Min.	Max.	SD	95% CI		
Mean																
I	10.90	139.44	137.16	140.00	98.00	179.00	14.70	13.07–16.81	141.72	145.00	100.00	195.00	18.25	15.48–22.21	0.140	0.030
II	11.90	149.36	146.60	148.00	110.00	184.00	13.84	12.30–15.82	152.13	152.00	115.00	215.00	19.20	16.29–23.37	0.550	0.010
Girls																
I	10.90	134.14	132.17	137.00	100.00	156.00	12.86	10.86–15.78	136.11	140.00	100.00	165.00	13.81	11.72–16.81	0.170	0.110
II	11.90	143.36	141.38	145.00	115.00	161.00	11.29	9.53–13.85	145.34	147.00	115.00	193.00	14.90	12.64–18.14	0.541	0.200
Boys																
I	10.90	144.74	142.16	145.00	98.00	179.00	14.71	12.55–17.75	147.32	147.00	105.00	195.00	15.70	13.33–19.21	0.301	0.050
II	11.90	155.37	151.83	153.00	110.00	184.00	14.08	12.02–16.99	158.91	155.00	115.00	215.00	19.22	16.32–23.41	0.610	0.020

GC—general education classes; SC—sport classes; measurement sessions: I (initial)—1–30 May-2018, II (final) 1–30 May 2019; Min.—minimum; Max.—maximum; SD—standard deviation; 95% CI—confidence interval; ESs—Effect sizes; * Mann-Whitney U Test; significant differences *p* ≤ 0.05.

**Table 3:** Results of the shuttle run test, sec.

Measurement Session	Average Age (y/o)	Total Average (sec.)	Group												ESs	*p* *
			GC						SC							
			Average	Median	Min.	Max.	SD	95% CI	Average	Median	Min.	Max.	SD	95% CI		
I	10.90	22.37	22.58	21.88	19.05	32.00	1.83	1.63–2.09	22.16	21.82	18.30	29.03	1.79	1.59–2.04	0.235	0.090
II	11.90	21.26	21.56	21.05	18.73	28.99	1.75	1.55–2.00	20.96	20.90	18.01	28.02	1.67	1.48–1.91	0.621	0.024
Girls																
I	10.90	22.83	23.03	22.60	20.50	30.35	1.70	1.44–2.09	22.64	22.00	19.59	29.03	1.74	1.56–2.24	0.410	0.230
II	11.90	21.78	22.02	21.89	18.82	28.02	1.85	1.56–2.28	21.55	21.50	18.05	25.92	1.82	1.54–2.21	0.580	0.300
Boys																
I	10.90	21.90	22.13	21.74	19.05	32.00	1.91	1.58–2.23	21.68	21.60	18.30	24.98	1.60	1.36–1.95	0.371	0.140
II	11.90	20.73	21.10	20.73	18.73	28.99	1.54	1.31–1.86	20.36	20.65	18.01	24.16	1.42	1.20–1.73	0.617	0.390

GC—general education classes; SC—sport classes; measurement sessions: I (initial)—1–30 May-2018, II (final) 1–30 May-2019; Min.—minimum; Max.—maximum; SD—standard deviation; 95% CI—confidence interval; ESs—Effect sizes; * Mann-Whitney U Test; significant differences *p* ≤ 0.05.

**Table 4:** Averaged fitness tests results from the initial and final measurement sessions.

Eurofit Tests	Group	Average	Median	Min.	Max.	SD	95% CI	*p* *	*p* **
10 × 5 m shuttle run (sec.)	SC	21.72	21.50	18.01	29.03	1.78	1.64–1.96	0.024	0.631
	GC	22.04	21.68	18.72	32.00	1.86	1.71–2.04		
standing long jump (cm)	SC	146.92	147.00	100.00	215.00	17.89	16.43–19.64	0.001	0.148
	GC	142.26	145.00	98.00	184.00	15.01	13.79–16.47		
sit-ups in 30 s (*n*/30 s)	SC	20.25	21.00	9.00	29.00	3.86	3.55–4.24	0.003	0.064

GC—general education classes; SC—sport classes; Min.—minimum; Max.—maximum; SD—standard deviation; 95% CI—confidence interval; *p* * = ANOVA; significant differences *p* ≤ 0.05; *p* **—test Levene’s Test *p* > 0.05.

**Table 5:** Averaged fitness test results from the initial and final measurement sessions in a group of girls and boys.

Variable	Class	Referenced	Average	Median	Min.	Max.	SD	95% CI	*p* *	*p* **
10 × 5 m shuttle run (sec.)	Girls	≥22.63	22.34	21.99	18.05	30.35	1.87	1.72–2.08	0.025	0.127
	Boys	≥21.78	21.45	21.30	18.01	32.00	1.68	1.54–1.84		
standing long jump (cm)	Girls	≥137.50	138.82	140.00	100.00	193.00	14.16	12.98–15.56	<0.001	0.521
	Boys	≥147.70	149.94	150.00	98.00	215.00	17.04	15.68–18.67		
sit-ups in 30 s (*n*/30 s)	Girls	≥17	18.61	19.00	8.00	27.00	3.50	3.21–3.85	<0.001	0.875
	Boys	≥18	20.57	21.00	2.00	29.00	3.83	3.52–4.19		

GC—general education classes; SC—sport classes; Min.—minimum; Max.—maximum; SD—standard deviation; 95% CI—confidence interval; *p* * = ANOVA; significant differences *p* ≤ 0.05; *p* **—Levene’s Test *p* > 0.05.

## Data Availability

Data is available upon request.
